# A Book of Detachable Diet Lists; for Albuminuria, Etc., Etc.

**Published:** 1895-04

**Authors:** 


A BOOK OF DETACHABLE DIET LISTS FOR ALBUMINURIA, ANAEMIA AND DEBILITY, CONSTIPATION, DIABETES, DIARRHOEA, DYSPEPSIA, FEVERS, GOUT OR URIC ACID, DEATHESIS, OBESITY, TUBERCULOSIS AND A SICK-ROOM DIETARY.—Compiled by Jerome B. Thomas, A. B., M. D., Visiting Physician to the Home for the Friendless Women and Children, and to the Newsboys Home; Assistant Visiting Physician to the King County Hospital, etc Price $1.50. Philadelphia: W. B. Saunders, 925 Walnut Street. 1895.

The title of this book gives a good idea of the contents There are ten different lists besides the sick-room dietary, and instructions for preparing many of the delicate and nutritious foods used in the sick-room. There are some fifteen or twenty detatchable leaves in each list intended to be detatched and placed in charge of the nurse. On each list there is blank space for special orders, for name of physician, for patient, etc. These lists will be found quite useful where patients are to be placed on special diet. They are "compiled from the most modern works on dietary," are convenient and ever ready for use, giving lucid directions for the preparation of food with which cooks are apt to be unfamiliar. V. H. T.

